# Agricultural Openness and the Risk of COVID-19 Incidence: Evidence from China

**DOI:** 10.3390/ijerph19063517

**Published:** 2022-03-16

**Authors:** Dezhen Wang, Buwajian Abula, Aniu Jizuo, Jianhua Si, Kaiyang Zhong, Yujiao Zhou

**Affiliations:** 1 College of Economics and Management, Xinjiang Agricultural University, Urumqi 830052, China; dezhengumei@163.com; 2 Business School, Yulin Normal University, Yulin 537000, China; 3 School of Public Policy & Management, Tsinghua University, Beijing 100084, China; jzan18@mails.tsinghua.edu.cn; 4 School of International Studies, Renmin University, Beijing 100872, China; jisi928@ruc.edu.cn; 5 School of Economic Information Engineering, Southwestern University of Finance and Economics, Chengdu 611130, China; zhongky@smail.swufe.edu.cn; 6 School of Economics, Southwestern University of Finance and Economics, Chengdu 611130, China

**Keywords:** griculture opening-up, input and transmission of COVID-19, entropy method, TOPSIS, grey correlation analysis

## Abstract

At present, there are large number of articles on the impact of COVID-19, but there are only a few articles on the impact of COVID-19 and international agriculture. Agriculture product is different from other industrial products. If domestic food cannot be self-sufficient, it must be resolved through imports. This will inevitably face the dilemma between the opening up agriculture and the risk of importing COVID-19. This paper pioneered the use of entropy method, TOPSIS method and grey correlation analysis to predict the correlation between agricultural opening to the outside world and the input and spread of COVID-19. We use the correlation matrix quantifying the number of confirmed COVID-19 cases and agricultural openness to deduce that there is a significant positive correlation between the flow of agricultural products caused by China’s agricultural opening-up and the spread of COVID-19, and use the proposed matrix to predict the spread risk of COVID-19 in China. The results of the empirical analysis can provide strong evidence for decision-makers to balance the risk of COVID-19 transmission with the opening of agricultural markets, and they can take this evidence into full consideration to formulate reasonable policies. This has great implications both for preventing the spread of COVID-19 and for agricultural opening-up.

## 1. Introduction

Agriculture and food security are important issues that need to be addressed and resolved globally, but this issue has become more serious due to the impact of COVID-19. According to the “State of Food Security and Nutrition in the World 2021 [1]” jointly issued by the Food and Agriculture Organization of the United Nations, the International Fund for Agricultural Development, United Nations International Children’s Emergency Fund, the United Nations World Food Program and the World Health Organization. By 2020, 720 million to 811 million people will still face hunger. It is equivalent to about 1/10 of the global population. The economic recession caused by the impact of COVID-19 has led to the largest increase in the number of hungry people in the world in decades, which affecting almost all low-income and middle-income countries. Conflicts, violent incidents and climate disasters in many parts of the world have also brought unfavorable factors to food security, coupled with long-standing inequality, food security and nutritional status issues that have affected people in many parts of the world.

In order to solve the problem of food security, in the past, countries have strengthened international cooperation in agriculture and promoted international trade in agriculture. According to a report issued by the Food and Agriculture Organization of the United Nations (State of Agricultural Commodity Markets 2020) [2], the global agricultural food trade has more than doubled since 1995, and the global trade volume reached USD 1.5 trillion in 2018. However, the COVID-19 epidemic is spreading throughout the world, posing great challenges to the supply, transportation and sales of agricultural products and the agricultural industry chain. How to ensure the sustainable and effective supply of agricultural products? What does the COVID-19 epidemic bring to the future development of agricultural production and the opening-up of agriculture? On the one hand, China is one of the largest importers of agricultural products in the world, with imports of agricultural products reaching USD 170.8 billion in 2020 (data from the official website of the General Administration of Customs of China) [3]; on the other hand, China has made great contributions to the world in fighting the COVID-19 epidemic, but it is also facing great pressure on COVID-19 epidemic prevention. Therefore, this paper will take China as an example for analysis.

Agricultural opening-up is to increase and promote agricultural imports and exports, expand domestic and foreign agricultural exchanges and cooperation. The current global situation of new crown epidemic prevention and control is not optimistic, and cold chain agricultural products have repeatedly become the transmission carrier of the virus entering China. From the outbreak of the first new crown epidemic in the South China Seafood Market to the effective control of the current epidemic in China, many of the outbreaks in various places are related to imported frozen fresh agricultural products. On 13 June 2020, the new crown virus was detected on the imported salmon cutting board at the Xinfadi Seafood Wholesale Market in Beijing. Many new patients in Beijing showed direct or indirect contact with the market. On 23 July 2020, the new coronavirus was detected in many food and environmental samples such as the processing workshop, dormitory, and canteen of Dalian Kaiyang Seafood Company. Many employees of the company and related imported aquatic products companies were confirmed to be infected with the new crown epidemic. On 12 August 2020, two samples of Brazilian frozen chicken whole wings submitted for inspection by the Longgang District Center for Disease Control and Prevention in Shenzhen tested positive for the new coronavirus nucleic acid, and so on. Various information shows that novel Coronavirus is highly correlated with the wholesale of live agricultural products, and there is a lot of evidence that each outbreak of COVID-19 was imported from abroad with live agricultural products as the medium [4]. The more open agriculture is, the more imports and exports of agricultural products, the faster the epidemic spreads and the greater the possibility of infection. Zhang et al. [5] believed that half of countries are at risk of import concentration of moderate and above (high and very high). Alikhanli [6] considered that the COVID19 pandemic reduces household and global incomes, leading to lower demand for imports. The sudden outbreak not only poses a great health threat to people, but also has a significant impact on agricultural production, sales, trade and other circulation links. The main contribution of this paper is to propose an analysis framework for analyzing the relationship between agricultural opening-up and COVID-19’s import risk. In empirical analysis, this paper will use the entropy method and the TOPSIS method to evaluate the agricultural openness, and apply the grey correlation analysis to predict the correlation between agricultural opening to the outside world and risk of COVID-19 brought by import. We believe that our analysis will provide a useful reference for how to promote world agricultural development and sustainable trade of agricultural products under the COVID-19 epidemic [7].

## 2. Literature Review

### 2.1. The Prediction of Novel Coronavirus Pneumonia

By using ecological niche modeling (ENM) to collect COVID-19 epidemic data and nine social and economic variables, Ren et al. predicted the potential risk areas infected with COVID-19 epidemic in some megacities [8]. Their research results indicated that ENM method could be used as an early prediction tool to identify potential COVID-19 infection risk areas on a small scale. On the basis of studying the correlation between daily average temperature (AT) and average relative humidity (ARH) and daily COVID-19 cases in 30 provinces of China from 20 January 2020 to 11 February 2020, Qi et al. fitted the generalized additive model (GAM) to quantify the specific provincial correlation between meteorological variables and daily cases infected with COVID-19 during the study period [9]. In accordance with the research of Mubarak and Zin, tourism and mass religious gatherings (MRG) may pose significant public health risks in the case of cross-border and intro-community transmission of infection [10]. Fan et al. established the SEIR dynamic model of COVID-19 epidemic with a latent period based on complex network theory [11]. At the same time, according to the relevant data of COVID-19 epidemic in China and some regions, they simulated the model parameters for different scenarios and predicted the inflection point of COVID-19 epidemic in three situations by setting three different virus latent periods. Sheng et al. used the classic SIR model (Susceptible Infected Recovered Model) and differential recursion method to analyze and predict the COVID-19 epidemic situation in the control stage on the basis of preprocessing the epidemic data of COVID-19 [12]. Assimilating model parameters were based on the new coronavirus pneumonia epidemic data released by the Municipal Health Commission. Yuan et al. not only used the “empirical data assimilation SARS case epidemic trend model method” to summarize the research team’s analysis and decision-making suggestions on the epidemic trend of China (except Hubei Province), Hubei Province (except Wuhan City) and Wuhan City from 3 to 28 February 2020, but it also provided three forecast curves as a COVID-19 epidemic prevention and control guidance line, so as to predict the development of the COVID-19 epidemic [13]. Based on the economic situation of China before and after SARS epidemic in 2003, Tu analyzed and predicted the impact of COVID-19 epidemic on China’s economy from two aspects of short-term effect and long-term effect, and put forward corresponding measures from the government and enterprise levels, respectively [14]. Taking COVID-19 epidemic data released by the official website of National Health Commission of the People’s Republic of China on 23 January 2020 as the initial value, Zhang et al. used the improved SIR model equation and Runge-Kutta method to simulate and calculate the relationship between the proportion of the four groups of people and the time change, and predicted the spread law of COVID-19 epidemic [15]. Moreover, they also compared the corresponding prediction results with the number of infected persons and cured persons as well as deceased persons at the actual time node.

The continuing spread of COVID-19 has exacerbated the global food supply chain crisis. As China is the largest grain importer, natural disasters, emergencies, anti-globalization trends and suppliers’ default are the main inducements leading to the interruption of the transnational grain supply chain in the context of a large number of import source countries. The vulnerability of China’s transnational food supply chain is mainly manifested in the following aspects: The integration ability of transnational food supply chain is not strong, the infrastructure is not complete, the mode of transnational transportation is relatively single and the source countries of imported grain are relatively concentrated [16]. The global pandemic has exerted varying degrees of impact on the supply and demand situation and import demand of different agricultural products in China in the short and long term, especially livestock products. The decline in domestic supply and the increase in compensatory consumer demand will promote the sustained growth of import demand in the long term [17]. The global panic caused by COVID-19 will trigger international investment capital to take the opportunity to speculate on grain prices, leading to the rise of grain prices, which will affect China’s grain prices and the rise of food prices, thus triggering a global food crisis and bringing food import risks to China [18]. In the post-epidemic period, countries will continue to introduce strict control measures, which will have a huge negative impact on the international trade of agricultural products, including the inevitable global economic recession, the reversal of the global supply and demand pattern of agricultural products, the great restriction of global agricultural trade and the sharp rise of global agricultural prices. For China, it is bound to face risks such as macroeconomic shocks, increasing instability in international trade of agricultural products, over-concentration of agricultural import and export markets, and increasing restrictions on the import and export of some agricultural products [19].

### 2.2. The Measurement Method of China’s Agricultural Openness

Rao et al. analyzed the correlation coefficient between the per capita income and the agricultural opening degree of each country, and the results showed that they had significant correlation, but it had very low correlation with agricultural growth rate [20]. Based on the particularity of agricultural production and trade mode, Xiong et al. assigned the subjective weight of 0.2, 0.7 and 0.1 to the distribution of agricultural product export dependence, agricultural product import dependence and agricultural capital market openness, respectively, to calculate the opening degree of China’s agricultural industry from 2001 to 2011 [21]. Xu et al. constructed the opening degree index of China’s agriculture from the two aspects of market openness of agricultural products and agricultural production factors, which used to calculate the opening degree of China’s agriculture from 2000 to 2011 [22]. Then they used the grey relational degree model to calculate the grey correlation degree, static and dynamic suitability evaluation index and the three index systems, including the living standard of rural residents, the international competitiveness of agriculture and the ability of agricultural sustainable development [23]. They concluded that China’s agricultural opening to the outside world as a whole is basically in a moderate state. Yang et al. constructed four indicators of agricultural product export dependence, agricultural product import dependence, element input and export dependence and element input import dependence from two aspects of agricultural input and output, and integrated the four indicators into a comprehensive agricultural opening degree index by using factor analysis method [24].

## 3. Construction of Agricultural Openness Measurement Index

### 3.1. Agricultural Openness

Basically, before 2014, the measurement method of agricultural openness in China simply developed to the stage of subjective weighting index method. The research of Xiong et al. [21] did not consider the impact of agricultural technology introduction and cooperation, materialized capital and related services closely related to agricultural production on agricultural openness. Further, the weighting of this research also reflected the limitation of being too subjective. Although Xu et al. [22] increased the flow of pesticides, fertilizers and seedlings on the basis of Xiong et al., the weighting of their research also showed subjectivity. Traditional analysis methods, such as subjective weighting method, are not only random, but also lacking of uniform weighting standards. Therefore, the reliability of the conclusions is doubtful. At present, scholars have seldom used this kind of method.

### 3.2. Agricultural Openness Measurement Index

Agricultural policies all over the world have their own characteristics and are very complex, so it is difficult to find a unified subjective weighting value of measuring the agricultural openness in the world. Further, the subjective arbitrariness of weight changes leads to poor effectiveness of the subjective weighting index method. Theoretical modeling is too complex to be used in agriculture. Comparatively, the objective weighting index method is more suitable for measuring the opening degree of agriculture. However, little research has been conducted to construct or use scientific and objective measurement index of agricultural openness. In order to avoid the subjective arbitrariness in most previous studies, this study adopts more scientific principal components, factors, and a cluster analysis method and entropy weight to measure and calculate the openness of China’s agriculture.

Based on the above research findings, the objective measurement indicators of agricultural openness can be constructed from three aspects: per capita output of major agricultural products, import and export trade of China’s agriculture, living standards and quality of life of the people. Specifically, the per capita output of major agricultural products can be measured with six indicators closely related to agricultural products, including per capita grain, milk, aquatic products, edible oil, pork, beef and mutton output. The living standard of income consumption can be measured by the per capita disposable income of urban residents and the per capita consumption expenditure of rural residents (Table 1 for details). The hypothesis is that if this element promotes the opening of agriculture, its effect is positive; if this element hinders the opening of agriculture, its effect is negative. In Table 1, the “Effect” column indicates the “+ (−)” means a positive (negative) index under the measurement method, and the larger (smaller) it is, the greater (lesser) the effect on agricultural openness. The measurement is the volume of trade between Chinese provinces and foreign countries. Table 1 lists the weights of the two columns, where the weights of the first column category are the sum of the subdivided category weights of the second column.

According to the availability of data, the statistics used in this study include agricultural import and export data, which include agricultural exports, agricultural imports, agricultural factor exports and agricultural factor imports. The data came from China Statistical Yearbook and Customs Statistics from 2008 to 2020. and statistics on COVID-19 epidemic in all provinces released on the official website of the National Health Commission (data as of 30 June 2020).

Based on the availability of data, the agricultural import and export statistics data in this paper only included the export value of agricultural products, import value of agricultural products, input and export value of agricultural factors, and the COVID-19 epidemic data released by the official website of National Health Commission of the People’s Republic of China. In addition, other types of data mainly originated from the China Statistical Yearbooks from the most recent 10 years.

## 4. Methods and Procedures for Measuring Agricultural Openness

First, we use the principal component factor analysis method to integrate and calculate the indicators of openness, then use the Entropy TOPSIS method to measure and evaluate, and then compare and analyze them.

### Measurement of Agricultural Openness by Principal Component Factor Analysis

According to the requirements of principal component factor analysis, these 12 variables are transformed into another group of unrelated variables (F_1_, F_2_, …, F_n_) by linear transformation. The total variance of variables remains unchanged in mathematical transformation. Meanwhile, the first variable has the largest variance, which is called the first principal component, while the second variable has the second largest variance and is irrelevant to the first variable, which is called the second principal component. However, the eigenvalues of retention factors should be greater than 1, until the variance of the accumulated eigenvalues of principal components reaches 70%. This is mainly a dimension reduction method to calculate the weight of each principal component.

Factor analysis method is used to give weight to the 12 indexes measured above. At the same time, all the indexes after weight coefficient correction are integrated into a comprehensive agricultural openness index. The theoretical basis of weighting by factor analysis lies in the internal relations among variables reflected by common factors, which exactly shows the relative influence degree of evaluation indicators on evaluation objects. Therefore, the factor load obtained after orthogonal rotation can be understood as the important coefficient of common factor to variable, which is consistent with the meaning of weight. By improving the factor analysis method for calculating openness, this paper firstly calculates the correlation matrix of 12 indexes in turn by using factor analysis program. Furthermore, according to the contribution of factor analysis and the corresponding index with accumulated contribution greater than 0.7, three principal factors are obtained (however, the eigenvalue of the retention factor should be greater than 1, and each empirical analysis just needs to retain three principal factors). After obtaining the contribution coefficient of the principal factor, the weight coefficient of each evaluation index can be calculated by Equation (1).

(1) $$\beta_{i}=\sum_{i}^{T}A_{\mathrm{ij}}F_{i}$$

where T represents the number of principal factors; A_ij_ represents the weight coefficient of the *i*-th principal factor to the *j*-th evaluation index, with its absolute value used to calculate; Fi represents the contribution coefficient of the *i*-th principal factor; β_i_ represents the weight coefficient of the *i*-th evaluation index.

The β_i_ obtained by Equation (1) cannot guarantee that the sum of all β_i_ is exactly equal to 1. This situation is basically applicable to the analysis of the single province, autonomous region and municipalities. However, in terms of the comparison of many provinces, autonomous regions and municipalities, the factor coefficients based on principal component analysis will be inconvenient to compare because of the data variation of provinces, autonomous regions and municipalities. In order to ensure the comparability among provinces, autonomous regions and municipalities, the method of Equation (1) is used to transform β_i_ again to ensure that the sum of the weight coefficients is equal to 1.

(2) $$W_{j}=\frac{\beta_{i}}{\sum_{j\;=1}^{12}\beta_{i}}$$

In which: W_j_ represents the revised weight coefficient of the *j*-th evaluation index.

(3) $$\mathrm{Agricultural}\;\mathrm{openness}=\sum W_{j}{*X}_{\mathrm{ij}}$$

The analysis was based on 11 years of statistics from 2009 to 2019. In total, 31 samples (representing 31 provinces, autonomous regions and municipalities except Hong Kong, Macao and Taiwan) were selected to participate in the analysis. The factors with eigenvalue greater than 1 are extracted and retained, and there are exactly three factors every year. The LR test chi-square value of the model is shown in Table 2. The *p* value (Prob > chi2) is 0.0000. The model is extremely significant. The cumulative variance contribution rate is more than 70% almost every year. The value of KMO test is greater than 0.7 in two years and 0.5334 in one year, while the results of the other seven years are all between 0.6 and 0.7, indicating that the effect of principal component factor analysis is ideal (see Table 2 for details). Generally, the sum value (i.e., it is greater than 1) of factor eigenvalues retained by the maximum variance orthogonal rotation analysis involved in principal component factor analysis is the same, and the cumulative variance contribution rate is also the same.

According to the principal component factor analysis method to calculate the measurement system, the agricultural openness is shown in Table 3. On the whole, the agricultural openness of most provinces, autonomous regions and municipalities showed a fluctuating upward trend from 2009 to 2018, indicating that due to the influence of the domestic and international environment, the trend of “going out and bringing in” of Chinese agriculture is obvious, and people’s living standards have improved significantly. In addition, we found that only 10 provinces and cities such as Beijing and Tianjin have positive scores, and the remaining 21 provinces and cities have negative scores. This shows that there are great differences in the development of agricultural economy between provinces. Different provinces have different degrees of agricultural opening, and agricultural opening has always been an important issue for the agricultural development of each province. Judging from the comprehensive ranking, Jiangsu, Guangdong, Shandong, Inner Mongolia and Shanghai ranked the top five in terms of agricultural opening. The characteristics of these provinces are: First, they are economically developed, relatively lack agricultural products, rely on imports and have strong foreign demand for agricultural products. Second, some provinces are large agricultural provinces and have been committed to the strategy of agricultural products going out to meet export demand. Hainan, Tibet, Jiangxi, Shanxi and Guizhou are ranked low in agricultural openness. These provinces are generally underdeveloped areas, with low economic development and per capita consumption levels, so import demand is small. Agriculture is relatively underdeveloped, so the demand for agricultural exports is relatively small. It can be seen that the ranking sequence of various provinces, autonomous regions and municipalities is very unstable in the first few years and the last few years of this decade. The change range relatively large. This shows that the economic structure of provinces, autonomous regions and municipalities has undergone great adjustment in the first few years and the last few years of the decade.

## 5. Calculation and Measurement of Agricultural Openness based on Entropy TOPSIS Method

If other methods are used for calculation, the loss of weight information will be relatively large. This also leads to the unsatisfactory measurement effect of the development level of agricultural openness. The entropy method makes full use of and excavates information from all data. In order to avoid the loss of information, it is necessary to avoid the interference and influence of human factors to a greater extent.

In order to analyze the contribution of each index for agriculture opening to the outside world, this study uses the entropy TOPSIS method to measure the development level of China’s regional agricultural opening-up, which was adopted by on Guo et al., Peng et al., Peng et al., Feng et al., Wei et al., Zhang et al., Sun et al. [25,26,27,28,29,30,31]. By contrast to the subjective assignment method to measure the degree of agricultural opening to the outside world, the entropy method has obvious advantages. As the agricultural opening degree measured by the entropy method, it not only includes the trend and information of the degree of agricultural opening calculated by any other analysis methods, but also it compares the degree of agricultural openness of each province and province-level municipality in China for each year, which is more gradual and stable and more in line with the law of economic development. The steps of this method are as follows:

The first step is to standardize the data before determining the weight due to the dimensional and unit differences among the indicators. Then, we use the range method to standardize the measurement index X_ij_ in the measurement system of agricultural development level open to the outside world:

(4) $$Y_{\mathrm{ij}}=\left\{\begin{array}{c} \frac{X_{\mathrm{ij}}-\mathrm{Min}\left(X_{\mathrm{ij}}\right)}{\mathrm{Max}\left(X_{\mathrm{ij}}\right)-\mathrm{Min}\left(X_{\mathrm{ij}}\right)},\;Xij\;Positive\;indicator\\ \frac{\mathrm{Max}\left(X_{\mathrm{ij}}\right)-X_{\mathrm{ij}}}{\mathrm{Max}\left(X_{\mathrm{ij}}\right)-\mathrm{Min}\left(X_{\mathrm{ij}}\right)},\;Xij\;Negative\;indicator \end{array}\right.$$

where i is province, j is measure index; X_ij_ and Y_ij_ are the original and standardized agricultural development level measurement index values, respectively; Max (X_ij_) and Min (X_ij_) are the maximum and minimum values of X_ij_.

The second step is to calculate the information entropy E_j_ of each measure index Y_ij_ in the measurement system of agricultural development level open to the outside world:

(5) $$E_{j}\;=\ln\frac{1}{n}\sum_{i\;=1}^{n}[(Y_{\mathrm{ij}}/\sum_{i\;=1}^{n}Y_{\mathrm{ij}})\ln\left(Y_{\mathrm{ij}}/\sum_{i\;=1}^{n}Y_{\mathrm{ij}})\right]\;(0\;\leq E_{J}\leq \;1)$$

The third step is to calculate the difference coefficient of j index. The degree of difference changes inversely with the entropy value. The smaller the entropy value, the greater the degree of difference between indexes, the greater the information reflected, and the greater the influence and effect of indexes on the evaluation objects.

(6) $$Fj_{\;}=1-E_{j}(j=1,\;2,\;\ldots ,\;m)$$

The fourth step is to determine the weight of j index and calculate the weight of Yij index in the measurement system of agricultural development level open to the outside world:

(7) $$W_{j}=f_{j}\;/\sum_{j\;=1}^{m}\mathrm{fj}$$

The fifth step is to construct the weighted matrix G for measuring the development level of agriculture opening to the outside world:

(8) $$G={\left(g_{\mathrm{ij}}\right)}_{nⅹm}$$

Among them: g_ij_ = W_j_ × Y_ij._

We determine the optimal scheme MAX^+^_j_ and the worst scheme MIN-_j_ according to the weighted matrix G:

MAX^+^_j_ = (maxr_i1_, maxr_i2_, …, maxr_ij_)

MIN^−^_j_ = (minr_i1_, minr_i2_, …, minr_ij_) (9)

Then, we calculate the Euclidean distance S^+^_i_ and S^−^_i_ between each measure scheme and the optimal scheme MAX^+^_j_ and the worst scheme Min^−^_j_:

(10) $$S_{i}^{+}=\sqrt{\sum_{j\;=1}^{m}{\left({\mathrm{MAX}}_{j}^{+}-g_{\mathrm{ij}}\right)}^{2}}$$

$$S_{i}^{-}=\sqrt{\sum_{j\;=1}^{m}{\left({\mathrm{MIN}}_{j}^{-}-g_{\mathrm{ij}}\right)}^{2}}$$

Lastly, we calculate the relative proximity between each measure scheme and the ideal scheme IDEAL_i_:

(11) $${\mathrm{IDEAL}}_{i}=\frac{S_{i}^{-}}{S_{i}^{+}+{\;S}_{i}^{-}}$$

Relative proximity IDEAL_i_ is between 0 ≤ IDEAL_i_ ≤ 1. When IDEAL_i_ = 0, G_i_= MIN-j, it indicates that the target is the worst target. When IDEAL_i_ = 1, G_i_= MAX^+^_j_, it indicates that the target is the optimal target. The greater the IDEAL_i_ value is, the higher the agricultural opening and development level of Province I is. On the contrary, the agricultural development level of province i is lower. In practical multi-objective decision making, the possibility of the optimal target and the worst target is very small.

Sorting the ideal solution according to IDEAL_i_ size:

According to the IDEAL_i_ values, the evaluation objectives are arranged from small to large. Sort results in the greater IDEAL_i_ value, the better the target, and the largest IDEAL_i_ value, which is the optimal bid evaluation target.

### 5.1. Weight Determination of Indexes

Under entropy method to calculate weight of the element layer as shown in Table 4, the import and export of agricultural products and agricultural inputs weights to reach 75%. If it is combined with the weight of income consumption and living standards, it reaches almost 90% This is also somewhat similar to most traditional methods for measuring the degree of agricultural opening to the outside world. Compared with the previous principal component factor analysis method in which the weights of each measurement index are equal, the statistical calculation accuracy and reliability are greatly improved.

Compared with the traditional method which uses the subjective assignment method to calculate and measure agricultural openness, the entropy method has obvious advantages. For instance, it can be clearly seen from Table 5 that the agricultural openness calculated and measured by the entropy method includes the trend and information of agricultural openness calculated and measured by other analysis methods. Moreover, compared with the agricultural openness of provinces, autonomous regions and municipalities in different years, it not only reflects the characteristics of gradual change and stability, but also accords with the development law of economic events.

### 5.2. Score and Ranking of Comprehensive Level of Agricultural Openness

Here is the comprehensive level of agricultural opening score rankings and confirmed cases of COVID-19 in all provinces and province-level municipalities of China. We calculate the maximum and minimum evaluation values of the development level of agricultural openness of China from 2009 to 2019 (Table 6). Then, we use the TOPSIS measurement scheme to calculate the MAX^+^_j_ value of the optimal scheme and the MIN^−^_j_ value of the worst scheme of each province and province-level municipality, and their respective Euclidean distances S^+^_i_ and S^−^_i_. Next, we calculate the approach degree of IDEAL_i_ of the ideal solution for each province and province-level municipality and arrange the specific values of them in ascending order according to the value of IDEAL_i_. The higher the IDEAL_i_ value is, the higher development level of agricultural openness of the province or province-level municipality is, and the highest IDEAL_i_ value is, the most ideal bid-evaluated agricultural openness level (Table 6).

From all over China, and observing the new leaders of confirmed cases of patients from statistical and news reports [4], you can see that agriculture as the external open area seems to be related to more cases of patients. By confirming the confirmed cases of new coronavirus patients in China’s provinces, autonomous regions and municipalities, one can observe it is related to agriculture, and the correlation is also large. We try to find some kind of contact from the following analysis. We rank the comprehensive level score of agricultural openness and confirmed cases of COVID-19 in all provinces. The data in the last column of Table 7 are the total number of confirmed COVID-19 cases in the provinces shown. Total confirmed cases of COVID-19 by province are shown in the column name. See Table 7 for details

**Figure 1:**
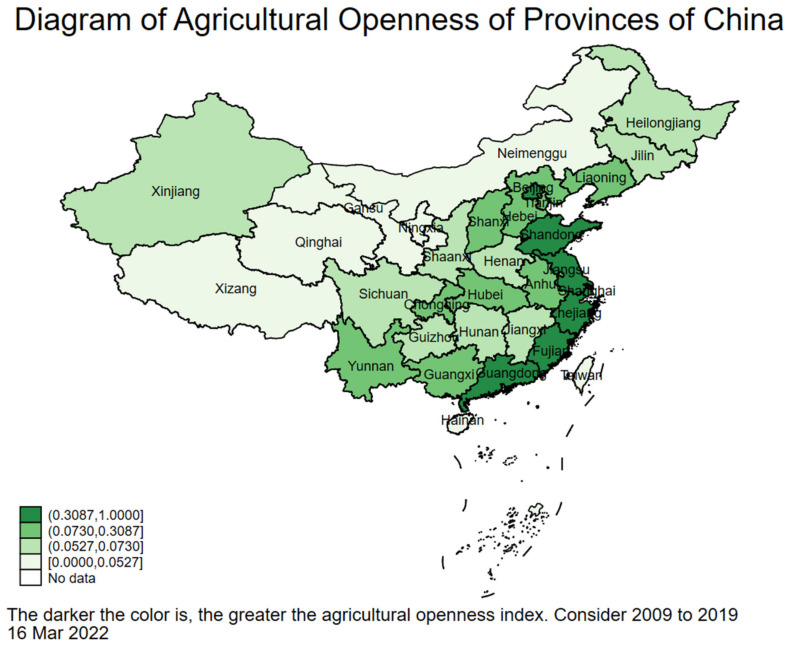
Comprehensive level of agricultural opening to the outside world.

According to the scores of the comprehensive level of agricultural opening-up in each province and province-level municipality in Table 7, the level of agricultural opening-up can be divided into four grades. In the first grade, the comprehensive level score of agricultural opening-up is 0.3087 < IDEAL ≤ 1.0000. In the second grade, it is 0.0730 < IDEAL ≤ 0.3087. The third grade of agricultural development comprehensive level score is 0.0527 < IDEAL ≤ 0.0730, In the fourth grade, the score is 0.0000 < IDEAL ≤ 0.0527. The IDEAL_i_ value of each province and province-level municipality decreases from left to right and from top to bottom (Table 8 for details). From the following analysis and Figure 1, it can be seen that the comprehensive level of agricultural opening-up are highly correlated with the confirmed cases of COVID-19 in all provinces and province-level municipalities in China. Therefore, Table 8 can also be regarded as COVID-19 imported and transmitted risk gradient table.

In order to visualize and compare the IDEAL value of each province and province-level municipality in China, this study draws a schematic diagram of the comprehensive level of agricultural opening up to the outside world from 2009 to 2019 (Figure 1). It can clearly show that the provinces and province-level municipalities in the first grade of the comprehensive score of agricultural opening to the outside world have the darkest colors. Except for Beijing and Shanghai, all the provinces and province-level municipalities in this grade are located in the economically developed coastal areas. Further, the provinces and province-level municipalities with the second grade are the second dark colors. In addition to Tianjin, Liaoning and Guangxi, provinces and province-level municipalities in this grade are also mostly located in central or eastern western regions. Additionally, the second grade can also be divided into three adjacent geographic regions: Tianjin, Liaoning, Shanxi and Hebei in the Bohai Rim economic Zone; Guangxi and Yunnan in the western border areas; and Hubei, Anhui and Chongqing in the Yangtze River Economic Belt. In particular, Hubei, Chongqing, Sichuan and Shaanxi have all developed in clusters, which has produced both aggregation and spillover effects. The provinces with the third grade are relatively linear, except for Xinjiang at this grade, most of them are staggered with the second grade of provinces and province-level municipalities. The colors of the provinces in the fourth grade are the lightest, except for Hainan, which is mostly the traditional pastoral area (Figure 1 for details).

From the following analysis and Figure 1, it indicates that the comprehensive level of agricultural opening-up index in each province and region is highly related to the confirmed cases of COVID-19. Therefore, Figure 1 can also be regarded as a schematic diagram of the risk of COVID-19 input and transmission.

### 5.3. Comparative Analysis of Principal Component Factor Analysis and Entropy TOPSIS Method

Through cluster analysis and entropy TOPSIS measurement analysis of the above principal component factor analysis and weighted average connection method, many table and graphs are generated. The above tables and graphs have many numerical values and great information. In order to better compare the statistical differences between principal component factor method and entropy TOPSIS method of agricultural openness measurement system, we perform cluster analysis based on the weighted average connection method on the total synthesis and ranking results of the last two columns in Table 6 and Table 7. Among them, the number at the bottom of the tree diagram of cluster analysis represents the order and ranking numbers of provinces, autonomous regions and municipalities in the first column of Table 6 and Table 7, which can be used for comparison and comparative study.

**Figure 2:**
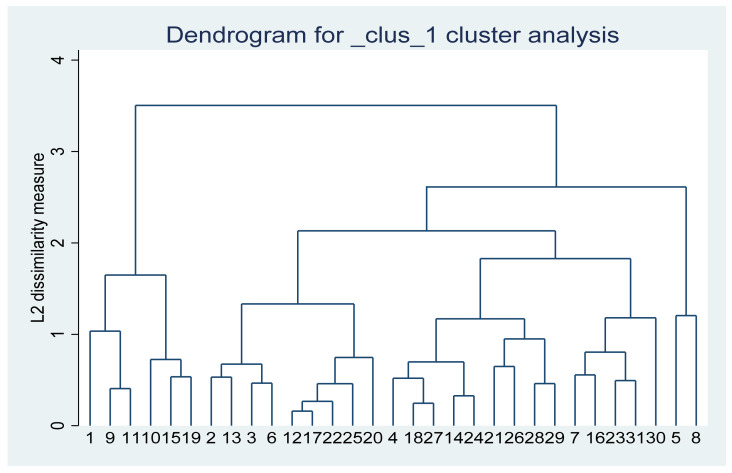
Tree diagram of clustering analysis based on weighted average connection method for the total synthesis and ranking results of agricultural openness.

It can be seen from Figure 2 that the samples with serial numbers 1, 9 and 11 represent Beijing, Shanghai and Zhejiang, respectively, while the samples with serial numbers 10, 15 and 19 represent Jiangsu, Shandong and Guangdong, respectively. These coastal areas with the most developed economy in China are clustered together first, because these provinces and municipalities mainly develop the tertiary industries including finance and education, as well as science and technology, with higher agricultural openness. Meanwhile, it also shows that these provinces and municipalities depend heavily on agricultural products. It can be clearly seen from the map in Figure 2. These provinces and municipalities are the first-class areas with the deepest color, with the agricultural openness index greater than 2.856.

Samples with serial numbers 2, 13, 3 and 6 represent Tianjin, Fujian, Hebei and Liaoning respectively, while samples with serial numbers 12, 17, 20, 22 and 25 represent Anhui, Hubei, Guangxi, Chongqing and Yunnan, respectively. These two types of areas are clustered together. Among these areas, some are economically developed coastal areas, while others are rapidly rising central areas or border areas in the last 10 years. Their common feature is that the rapid development of the secondary and tertiary industries leads to the gradual reduction of the proportion of the primary industry and the gradual increase of agricultural openness as well as the increasing dependence on agricultural products. It can be clearly seen from the map in Figure 2 that these provinces, autonomous regions and municipalities are almost the second-class areas with the deepest color, and the agricultural openness index is between 1.195 and 2.856.

Samples with serial numbers 4, 18, 27, 14 and 24 represent Shanxi, Hunan, Shaanxi, Jiangxi and Guizhou, respectively, while samples with serial numbers 21, 26, 28 and 29 represent Hainan, Tibet, Gansu and Qinghai, respectively. These two types of areas are clustered together. Most of these areas belong to traditional mountainous agricultural areas or animal husbandry areas, which have excellent ecological barriers and low agricultural openness as well as small dependence on agricultural products. Samples with serial numbers 7, 16, 23, 31 and 30 represent Jilin, Henan, Sichuan, Xinjiang and Ningxia, which are clustered together. These areas belong to traditional agricultural self-sufficiency areas, with a large proportion of agriculture. For example, the agricultural openness in Xinjiang has gradually increased in recent 10 years. Cotton production alone currently accounts for almost 80% of the whole country. In addition, in recent years, Xinjiang has vigorously developed other agricultural and livestock production. Samples with serial numbers 5 and 8 represent Inner Mongolia and Heilongjiang. These two provinces are quite special. Among them, as the area where traditional agricultural and animal husbandry areas are equally important, one province focuses on the development of animal husbandry, while the other focuses on the development of agriculture, with low agricultural openness and less dependence on agricultural products. It can be clearly seen from Figure 2 that Tibet, Gansu, Qinghai, Ningxia and Inner Mongolia are the lightest areas with the smallest agricultural openness and the smallest dependence on agricultural products. At the same time, these areas are also ecological protection sites and water conservation sites in China. Although most of them are traditional animal husbandry areas, the state still advocates the policies of pastureland rehabilitation and forest rehabilitation from slope agriculture.

## 6. Risk Prediction of COVID-19 Input Transmission and Empirical Analysis of the Grey Correlation of Agricultural Openness of China

The outbreak of COVID-19 in 2020, which involves six continents, is still raging and has become the focus of global public health attention. For these countries or regions, it is urgent to implement effective strategies as soon as possible to curb the growing epidemic. Many studies have predicted the size and duration of potential COVID-19 outbreaks early to support the use of various models to develop effective infection prevention and control strategies in China and other countries. However, the identification of potential risk areas or areas of COVID-19 and its influencing factors is also of great significance for health departments to implement effective large-scale prevention and control measures. However, previous studies have rarely involved. Various information shows that the epidemic situation is highly correlated with the wholesale market of agricultural products, and many evidences show that the subsequent outbreaks of COVID-19 in China came from abroad with agricultural products as the medium [4,5,6]. How to find the connection between them?

The input and spread of COVID-19 and the opening up of agriculture can serve as a system of common development and change. Gray correlation analysis is a quantitative analysis to measure the development trend of this dynamic process which is the quantitative comparative analysis of the development trend. Next, this paper measures the degree of correlation between COVID-19 import, transmission and agricultural openness factors through the data on confirmed cases of COVID-19 published on the official website of the National Health Commission (data as of 30 June 2020). Taking China’s 2009–2018 agricultural opening-up comprehensive level sequence (see Table 7 for the IDEALi project sequence) and the 10 2009–2018 agricultural opening-up index sequences as comparison sequences, the entropy method was used to calculate the measurement system (Table 5).

This study draws on the grey relational analysis method which is used in the research of Li et al., Zhang et al. Bao et al., Yang et al. Zhong et al. [31,32,33,34,35,36], etc. The specific steps are as follows:

We take the number of confirmed cases of COVID-19 in each province and province-level municipality as a reference series:

Yi = (y_1_, y_2_, y_3_, ..., y_i_) i = 1,2,3, ...,31 (12)

The score series of the comprehensive level of agricultural openness and the 10-year series of agricultural opening-up indicators of each province and region are used as the compared sequence:

x_i_ = (x_i1,_ x_i2_, ..., x_ij_) i =1, 2, 3, ..., 31; j = 1,2, 3, ..., 11 (13)

Therefore, the correlation coefficient is defined as:

(14) $$\xi_{\mathrm{ij}}(k)=\frac{\mathrm{minmin}\left|\mathrm{xjk}-\mathrm{xik}\right|+\;\rho \mathrm{maxmax}\left|\mathrm{xjk}-\mathrm{xik}\right|}{\left|\mathrm{xjk}-\mathrm{xik}\right|+\;\rho \mathrm{maxmax}\left|\mathrm{xjk}-\mathrm{xik}\right|}$$

ρ ∈ (0,1), is the discrimination coefficient, generally take 0.5. Since ξ_ij_(k) only reflected the linear relationship of the points, the dispersion of the correlation information ξ_ij_(k) was needed to integrate with the depiction of the correlation of the serie [37]. The degree of correlation was defined as:

(15) $$r_{\mathrm{ij}}=\frac{1}{11}\sum_{k\;=1}^{11}\xi \mathrm{ij}\left(k\right)$$

**Figure 3:**
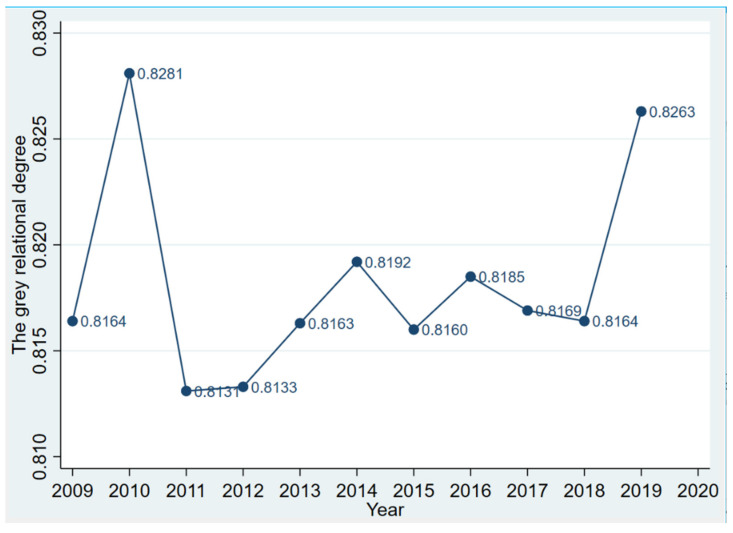
The grey correlation between confirmed COVID-19 cases in all provinces and municipalities and agricultural development over the years.

When|r_ij_| is greater than 0.7, it exhibits strong correlation; when it is less than 0.3, it exhibits weak correlation (Zhuo Jinwu, 2014). By calculating the gray correlation between the confirmed COVID-19 cases in each province, region and city and the agricultural opening index, the results are shown in Figure 3.

According to Figure 3, the comprehensive score of COVID-19 and the grey correlation of agricultural openness in all provinces and province-level municipalities of China exceeds 0.81 from 2009 to 2019, indicating a strong correlation between the confirmed COVID-19 cases and agricultural openness. Therefore, agricultural openness indicators can be used to predict and prevent the risk of COVID-19 import transmission everywhere.

## 7. Conclusions

At present, the novel coronavirus outbreak is spreading all over the world, posing great challenges to the supply and distribution of agricultural products. No matter in the production end, circulation or sales, the epidemic has an impact on agricultural production. In addition to the cultivation end, the agricultural industrial chain is long, from processing to channels, from retail, consumption to capital, including the diversified professional division of labor of commercialization, marketization and branding. Any problem in any link will cause a chain reaction. The opening up of agriculture can help a country make full use of both international and domestic markets, enrich the supply of domestic agricultural products and relieve the pressure on resources. However, people are eating food that seems delicious, diseases are entering into people’s mouths, and COVID-19 is spreading around the world, where every indication is that it will not go away completely in the next few years.

From the above empirical analysis, it can be seen that there is a high statistical link between the source, import and spread of COVID-19 and the import and openness of agricultural products of various provinces in China. The risk of outbreak and spread is high in areas with a high degree of agricultural openness, which should be a revelation of this study. This provides a statistically quantifiable direction for the importation, transmission and prevention of COVID-19 in China and other countries. It has strong pertinence and helps to save a lot of manpower, material and financial resources. Although the opening-up of agriculture can promote local economic development and improve people’s lives, in order to prevent the importation and spread of the epidemic, inspection, quarantine, monitoring and management of agricultural products, especially fresh agricultural products, should be strengthened. Combined with the further development of agriculture in the future and people’s daily life, this study has important practical significance. At the same time, the COVID-19 risk prediction and prevention and control experience can not only be used for reference in other countries and regions, but also provide reference and basis for other countries and regions to develop modern agriculture and formulate agricultural opening-up policies.

This paper also has certain study limitations. The opening of confirmed cases in China’s provinces and cities to the outside world is closely related to agriculture. We try to find some connection. So, we only explored the correlation between agricultural openness and the new crown epidemic from the perspective of agricultural economy. We ignore the impact of other control variables on the new crown epidemic. Our next research will start from the general economic openness, conduct a comparative analysis with the agricultural openness, and combine other relevant factors to carry out the risk analysis of the new crown epidemic.

## Figures and Tables

**Table 1:** Agricultural openness measurement system.

Standard Level	Element Layer	Element Layer Weight (%)	Measurement Index	Index Measurement Unit	X_ij_	Effect	Measurement Index Weight (%)
Per capita output of main agricultural products	Basic living security	0.0302	Other grain production per capita	Kg	X_1_	-	0.0155
			Per capita cereal production	Kg	X_2_	-	0.0147
	quality of life Improvement	0.0486	Per capita edible oil production	Kg	X_3_	-	0.0209
			Per capita production of pork, beef and mutton	Kg	X_4_	-	0.0277
		0.0260	Per capita output of aquatic products	Kg	X_5_	-	0.0155
			Milk production per capita	Kg	X_6_	-	0.0105
Agricultural import and export	Import and export of agricultural products	0.4080	Export volume of agricultural products	$	X_7_	+	0.2129
			Imports of agricultural products	$	X_8_	+	0.1951
	Import and export of agricultural elements	0.3377	Agricultural factor input and export	$	X_9_	+	0.1404
			Agricultural factor input imports	$	X_10_	+	0.1973
Quality of living standard	Income and consumption standard	0.1495	Per capita disposable income of urban residents	Yuan	X_11_	+	0.1001
			Per capita consumption expenditure of rural residents	Yuan	X_12_	+	0.0494

**Table 2:** Analysis of principal component factors in agricultural openness measurement system.

	Item	Sum Value of Retained Factor Eigenvalues	Cumulative Variance Contribution Rate	Total Value of KMO Tests	LRtest Chi-Square Value	*p* Value
Year						
2019		8.3956	0.7118	0.7206	236.73	0.0000
2018		8.5477	0.7123	0.7211	238.68	0.0000
2017		8.4988	0.7082	0.7126	235.69	0.0000
2016		8.4418	0.7035	0.6826	221.16	0.0000
2015		8.3089	0.6924	0.6759	214.29	0.0000
2014		8.4962	0.7080	0.6590	229.56	0.0000
2013		8.5538	0.7128	0.6659	230.84	0.0000
2012		8.6616	0.7218	0.6413	267.67	0.0000
2011		8.6629	0.7219	0.6331	273.72	0.0000
2010		8.8194	0.7349	0.5334	395.59	0.0000
2009		8.4136	0.7011	0.6798	244.32	0.0000

**Table 3:** Statistical results of calculating agricultural openness by principal component factor method of measurement system.

	Year	2009	2010	2011	2012	2013	2014	2015	2016	2017	2018	2019	Synthesis	Ranking
Regions														
Beijing		0.472	−0.513	0.428	0.423	0.439	0.432	0.489	0.439	0.629	0.715	0.718	0.614	8
Tianjin		0.119	−0.426	0.146	0.221	0.266	0.255	0.259	0.240	−0.114	−0.053	−0.136	0.427	9
Hebei		−0.091	0.029	−0.071	−0.080	−0.037	−0.035	−0.012	−0.027	−0.095	−0.096	−0.141	0.347	12
Shanxi		−0.273	−0.574	−0.249	−0.209	−0.243	−0.234	−0.270	−0.289	−0.492	−0.434	−0.533	0.181	28
Neimenggu		0.559	0.798	0.652	0.632	0.687	0.628	0.678	0.697	0.698	0.648	0.779	0.805	2
Liaoning		−0.097	0.078	−0.085	−0.101	−0.109	−0.122	−0.124	−0.144	−0.168	−0.213	−0.169	0.312	14
Jilin		−0.224	0.003	−0.015	−0.021	−0.054	−0.065	−0.077	−0.081	0.007	−0.092	−0.063	0.346	13
Heilongjiang		0.353	0.416	0.530	0.471	0.409	0.393	0.272	0.299	0.543	0.556	0.554	0.653	7
Shanghai		0.763	−0.028	0.640	0.628	0.574	0.625	0.660	0.645	0.753	0.874	0.695	0.726	6
Jiangsu		0.907	0.946	0.895	0.908	0.937	0.878	0.870	0.962	1.035	1.061	1.028	0.939	1
Zhejiang		0.726	0.270	0.644	0.633	0.646	0.635	0.611	0.622	0.576	0.568	0.603	0.732	5
Anhui		−0.299	−0.051	−0.269	−0.254	−0.241	−0.224	−0.182	−0.149	−0.154	−0.130	−0.106	0.271	17
Fujian		0.020	−0.152	−0.010	−0.024	−0.019	−0.043	−0.065	−0.131	−0.037	−0.068	−0.004	0.351	11
Jiangxi		−0.391	−0.221	−0.387	−0.405	−0.388	−0.380	−0.354	−0.370	−0.386	−0.401	−0.376	0.156	29
Shandong		0.621	0.883	0.742	0.756	0.676	0.751	0.580	0.544	0.629	0.508	0.602	0.789	3
Henan		−0.260	0.389	−0.270	−0.272	−0.248	−0.236	−0.171	−0.182	−0.192	−0.221	−0.190	0.293	15
Hubei		−0.357	0.030	−0.320	−0.329	−0.273	−0.260	−0.153	−0.130	−0.096	−0.144	−0.047	0.276	16
Hunan		−0.330	−0.045	−0.365	−0.368	−0.331	−0.311	−0.298	−0.281	−0.276	−0.274	−0.219	0.213	23
Guangdong		0.824	1.281	0.700	0.717	0.695	0.690	0.719	0.715	0.462	0.404	0.377	0.784	4
Guangxi		−0.226	−0.339	−0.276	−0.238	−0.279	−0.302	−0.312	−0.360	−0.212	−0.251	−0.326	0.206	24
Hainan		−0.473	−0.444	−0.544	−0.593	−0.626	−0.643	−0.651	−0.695	−0.642	−0.660	−0.575	0.024	31
Chongqing		−0.147	−0.228	−0.152	−0.161	−0.211	−0.222	−0.259	−0.212	−0.188	−0.167	−0.199	0.260	18
Sichuan		−0.220	0.065	−0.321	−0.313	−0.289	−0.292	−0.281	−0.243	−0.165	−0.169	−0.135	0.255	19
Guizhou		−0.332	−0.410	−0.315	−0.318	−0.370	−0.334	−0.338	−0.288	−0.271	−0.287	−0.293	0.184	27
Yunnan		−0.134	−0.285	−0.176	−0.211	−0.267	−0.298	−0.265	−0.274	−0.179	−0.104	−0.068	0.255	20
Xizang		−0.395	−0.288	−0.491	−0.500	−0.469	−0.440	−0.402	−0.404	−0.383	−0.385	−0.386	0.132	30
Shaanxi		−0.242	−0.442	−0.232	−0.214	−0.242	−0.241	−0.248	−0.258	−0.405	−0.363	−0.434	0.199	25
Gansu		−0.285	−0.341	−0.272	−0.253	−0.255	−0.273	−0.313	−0.294	−0.353	−0.317	−0.365	0.195	26
Qinghai		−0.249	−0.079	−0.363	−0.357	−0.276	−0.276	−0.280	−0.252	−0.288	−0.315	−0.318	0.214	22
Ningxia		−0.048	−0.153	0.042	0.058	0.062	0.121	0.084	0.086	−0.003	0.053	−0.027	0.399	10
Xinjiang		−0.290	−0.169	−0.236	−0.224	−0.164	−0.177	−0.166	−0.181	−0.232	−0.244	−0.250	0.253	21

**Table 4:** Weight of entropy value method of agricultural opening.

	Item	Basic Life Guarantee Layer	Level of Quality Life Improvement	Improved The Quality Of Life	Agricultural Product Import and Export	Import and Export of Agricultural Inputs	Income Consumption Standard of Living
Year							
2009		0.0296	0.0448	0.0239	0.4149	0.3284	0.1585
2010		0.0281	0.0463	0.0222	0.3742	0.3919	0.1373
2011		0.0291	0.0582	0.0243	0.4233	0.3356	0.1295
2012		0.0330	0.0613	0.0242	0.4212	0.3334	0.1269
2013		0.0383	0.0611	0.0236	0.4009	0.3279	0.1481
2014		0.0304	0.0529	0.0237	0.3940	0.3444	0.1546
2015		0.0346	0.0504	0.0242	0.4148	0.3144	0.1616
2016		0.0313	0.0398	0.0246	0.4175	0.3236	0.1632
2017		0.0259	0.0418	0.0339	0.4116	0.3245	0.1624
2018		0.0260	0.0427	0.0319	0.4107	0.3349	0.1537
2019		0.0261	0.0352	0.0296	0.4051	0.3354	0.1486
Mean		0.0302	0.0486	0.0260	0.4080	0.3377	0.1495

**Table 5:** Statistical results of calculating agricultural openness by the entropy method of the measurement system.

	Year	2009	2010	2011	2012	2013	2014	2015	2016	2017	2018	2019	Synthesis	Rank
Regions														
Beijing		0.295	0.300	0.314	0.313	0.345	0.322	0.346	0.314	0.607	0.587	0.630	0.476	6
Tianjin		0.270	0.243	0.293	0.329	0.330	0.295	0.307	0.294	0.245	0.252	0.248	0.308	8
Hebei		0.209	0.156	0.215	0.210	0.220	0.202	0.225	0.210	0.220	0.207	0.185	0.195	11
Shanxi		0.126	0.113	0.133	0.141	0.144	0.128	0.133	0.116	0.109	0.106	0.095	0.075	16
Neimenggu		0.100	0.086	0.102	0.096	0.099	0.112	0.128	0.121	0.118	0.113	0.108	0.053	26
Liaoning		0.248	0.201	0.262	0.274	0.275	0.276	0.267	0.250	0.213	0.203	0.182	0.250	9
Jilin		0.131	0.099	0.129	0.137	0.130	0.112	0.116	0.104	0.095	0.102	0.089	0.063	23
Heilongjiang		0.140	0.099	0.129	0.136	0.136	0.114	0.103	0.096	0.123	0.119	0.110	0.069	18
Shanghai		0.443	0.537	0.447	0.434	0.452	0.436	0.444	0.436	0.686	0.678	0.604	0.630	5
Jiangsu		0.750	0.628	0.742	0.731	0.743	0.681	0.715	0.736	0.754	0.776	0.665	0.894	2
Zhejiang		0.556	0.560	0.579	0.570	0.585	0.546	0.551	0.546	0.618	0.610	0.564	0.728	4
Anhui		0.119	0.104	0.135	0.144	0.148	0.138	0.158	0.160	0.183	0.187	0.180	0.117	14
Fujian		0.284	0.264	0.311	0.328	0.338	0.317	0.323	0.304	0.385	0.372	0.347	0.370	7
Jiangxi		0.100	0.103	0.112	0.117	0.124	0.114	0.126	0.117	0.121	0.117	0.112	0.061	24
Shandong		0.630	0.424	0.733	0.721	0.688	0.693	0.645	0.620	0.586	0.544	0.502	0.753	3
Henan		0.111	0.089	0.124	0.122	0.126	0.112	0.129	0.120	0.134	0.121	0.112	0.067	19
Hubei		0.112	0.096	0.157	0.149	0.152	0.144	0.195	0.185	0.173	0.173	0.169	0.125	13
Hunan		0.107	0.096	0.108	0.114	0.124	0.116	0.118	0.120	0.136	0.145	0.152	0.073	17
Guangdong		0.725	0.881	0.723	0.745	0.746	0.705	0.742	0.741	0.650	0.592	0.531	0.894	1
Guangxi		0.220	0.125	0.219	0.264	0.255	0.224	0.221	0.202	0.280	0.248	0.195	0.224	10
Hainan		0.098	0.088	0.103	0.105	0.105	0.098	0.102	0.094	0.099	0.100	0.104	0.039	27
Chongqing		0.116	0.102	0.141	0.138	0.125	0.118	0.119	0.115	0.142	0.140	0.132	0.079	15
Sichuan		0.110	0.095	0.108	0.110	0.112	0.104	0.109	0.106	0.126	0.126	0.128	0.058	25
Guizhou		0.119	0.082	0.150	0.132	0.109	0.108	0.123	0.115	0.118	0.116	0.108	0.067	21
Yunnan		0.175	0.106	0.197	0.170	0.160	0.139	0.178	0.146	0.171	0.199	0.184	0.140	12
Xizang		0.075	0.073	0.074	0.081	0.081	0.071	0.074	0.071	0.072	0.079	0.077	0.004	31
Shaanxi		0.114	0.109	0.124	0.132	0.131	0.114	0.121	0.111	0.122	0.118	0.111	0.067	20
Gansu		0.092	0.081	0.094	0.096	0.093	0.087	0.088	0.081	0.092	0.089	0.083	0.024	29
Qinghai		0.071	0.068	0.072	0.074	0.081	0.078	0.081	0.078	0.089	0.084	0.081	0.010	30
Ningxia		0.098	0.088	0.096	0.101	0.099	0.089	0.090	0.083	0.087	0.086	0.078	0.027	28
Xinjiang		0.116	0.110	0.115	0.125	0.132	0.114	0.116	0.109	0.121	0.115	0.109	0.064	22
Average		0.221	0.200	0.234	0.237	0.238	0.223	0.232	0.223	0.248	0.242	0.225		

**Table 6:** The extreme value table of 2009–2018 agricultural openness value.

	Years	2009	2010	2011	2012	2013	2014	2015	2016	2017	2018	2019
Extremum												
MAX		0.7499	0.8809	0.7423	0.7448	0.7464	0.7052	0.7419	0.7414	0.7537	0.7757	0.6646
MIN		0.0714	0.0683	0.0718	0.0743	0.0808	0.0707	0.0737	0.0711	0.0724	0.0790	0.7671

**Table 7:** The ranking of the comprehensive level score of agricultural openness and confirmed cases of COVID-19 in all provinces.

Provinces (Cities)	S^+^_i_	S^−^_i_	Ideal	Rank	Total Confirmed Cases of COVID-19 by Province
Beijing	1.2730	1.1555	0.4758	6	919
Tianjin	1.5656	0.6991	0.3087	8	198
Hebei	1.8167	0.4407	0.1952	11	349
Shanxi	2.0882	0.1687	0.0747	16	198
Neimenggu	2.1384	0.1189	0.0527	26	238
Liaoning	1.7012	0.5657	0.2496	9	155
Jilin	2.1185	0.1413	0.0625	23	155
Heilongjiang	2.1007	0.1564	0.0693	18	947
Shanghai	0.8690	1.4774	0.6297	5	712
Jiangsu	0.2560	2.1481	0.8935	2	654
Zhejiang	0.6164	1.6523	0.7283	4	1269
Anhui	2.0000	0.2659	0.1173	14	991
Fujian	1.4288	0.8395	0.3701	7	363
Jiangxi	2.1134	0.1377	0.0612	24	932
Shandong	0.6002	1.8266	0.7527	3	792
Henan	2.1031	0.1515	0.0672	19	1276
Hubei	1.9860	0.2840	0.1251	13	68135
Hunan	2.0937	0.1649	0.0730	17	1019
Guangdong	0.2515	2.1228	0.8941	1	1641
Guangxi	1.7656	0.5107	0.2243	10	254
Hainan	2.1673	0.0866	0.0385	27	171
Chongqing	2.0763	0.1783	0.0791	15	582
Sichuan	2.1227	0.1303	0.0597	25	595
Guizhou	2.1099	0.1509	0.0668	21	147
Yunan	1.9488	0.3167	0.1398	12	185
Xizang	2.2434	0.0087	0.0039	31	1
Shaanxi	2.0998	0.1509	0.0670	20	320
Gansu	2.1985	0.0529	0.0235	29	164
Qinghai	2.2349	0.0215	0.0095	30	18
Ningxia	2.1927	0.0607	0.0269	28	75
Xinjiang	2.1066	0.1435	0.0638	22	76

**Table 8:** Classification of comprehensive level of agricultural opening-up of each province.

Min < IDEAL_i_ ≤ Max	Region Classification (IDEAL_i_ Values Decrease from Left to Right, Top to Bottom)
0.3087 < IDEAL_i_ ≤ 1.0000	Guangdong, Jiangsu, Shandong, Zhejiang, Shanghai, Beijing, Fujian
0.0730 < IDEAL_i_ ≤ 0.3087	Tianjin, Liaoning, Guangxi, Hebei, Yunnan, Hubei, Anhui, Chongqing, Shanxi
0.0527 < IDEAL_i_ ≤ 0.0730	Hunan, Heilongjiang, Henan, Shaanxi, Guizhou, Xinjiang, Jilin, Jiangxi, Sichuan
0.0000 < IDEAL_i_ ≤ 0.0527	Neimenggu, Hainan, Ningxia, Gansu, Qinghai, Xizang

## Data Availability

Not applicable.

## References

[1] The State of Food Security and Nutrition in the World 2021. https://www.fao.org/publications/sofi/en/.

[2] State of Agricultural Commodity Markets 2020. https://www.fao.org/resources/digital-reports/state-of-agricultural-commodity-markets/en.

[3] The General Administration of Customs of China. http://www.customs.gov.cn.

[4] National Health Commission of the People’s Republic of China. http://www.nhc.gov.cn.

[5] Zhang C., Yang Y., Feng Z., Xiao C., Liu Y. (2021). Risk of global external cereals supply under the background of the COVID-19 pandemic: Based on the perspective of trade network. Foods.

[6] Alikhanli Y. (2020). Some scenarios on the impacts of the COVID-19 pandemic to automobile import demand function in azerbaijan. J. Phys. Conf. Ser..

[7] Liu Y., Yang Y., Li H., Zhong K. (2022). Digital economy development, industrial structure upgrading and green total factor productivity: Empirical evidence from China’s cities. Int. J. Environ. Res. Public Health.

[8] Ren H., Zhao L., Zhang A., Song L., Liao Y., Lu W., Cui C. (2020). Early forecasting of the potential risk zones of COVID-19 in China’s megacities. Sci. Total Environ..

[9] Qi H., Xiao S., Shi R., Ward M.P., Chen Y., Tu W., Su Q., Wang W., Wang X., Zhang Z. (2020). COVID-19 transmission in mainland China is associated with temperature and humidity: A time-series analysis. Sci. Total Environ..

[10] Mubarak N., Zin C.S. (2020). Religious tourism and mass religious gatherings—The potential link in the spread of COVID-19. Current perspective and future implications. Travel Med. Infect. Dis..

[11] Fan R., Wang Y., Lin J. (2021). Study on multi-agent evolutionary game of emergency management of public health emergencies based on dynamic rewards and punishments. Int. J. Environ. Res. Public Health.

[12] Sheng H., Wu L., Xiao C. (2020). Modeling analysis and prediction of COVID-19 transmission. J. Syst. Simul..

[13] Yuan H., Liang M., Huang Q., Su G., Chen T., Chen J., Sun Z., Yang S., Deng L., Li K. (2020). Application of case method of empirical data assimilation in COVID-19 epidemic analysis. Sci. Tec. Rev..

[14] Tu T. (2020). Prediction of the impact of COVID-19 on China’s economy and countermeasures. Market Res..

[15] Zhang Y., Liu W., Kong L., Xie C., Liu R., Hou R., Zhang M., Liu Y., Zhang Y., Hao B. (2020). Prevention of COVID-19 in stomatological hospitals (PART I). J. Appl. Stomatol..

[16] Sun H., Zhao Y. (2020). Research on the optimization of China’s transnational food supply chain based on crisis response. Economist.

[17] Cao L., Hong W., Zang B. (2021). China’s agricultural imports under the background of COVID-19 and the PHASE-1 agreement between China and the United States: Exogenous shocks and reasonable responses. Int. Trade.

[18] Wang X. (2020). Impact of COVID-19 on grain import in China and countermeasures. J. Wuhan Univ. Light Ind..

[19] Yang S., Wang W. (2021). International trade of agricultural products in the post-epidemic period: Risks and countermeasures. Price Mon..

[20] Rao B.B., Rao M. (2009). Openness and growth in Fiji: Sometime series evidence. Appl. Econ..

[21] Xiong Q., Wen S. (2012). Indicators and measurement of China’s agricultural openness to the outside world: 1997–2011. Reform.

[22] Xu R., Chen Y., Wang B. (2014). Measurement and evaluation of China’s agricultural openness to the outside world—From the perspective of agricultural development. S. Rural..

[23] Zhong K.Y. (2022). Does the digital finance revolution validate the environmental Kuznets curve? Empirical findings from China. PLoS ONE.

[24] Yang S., Xiong Q. (2015). Measurement and international comparison of China’s agricultural openness to the outside world: 1991–2011. Rural Econ..

[25] Guo X., Chen S. (2014). Remeasurement of economic openness and China’s economic growth. E. China Econ. Manag..

[26] Peng D., Liu C., Song Y. (2016). Evaluation of new urbanization level and Spatio-temporal evolution analysis of the Yangtze River economic belt. Stat. Decis..

[27] Peng D., Chen B., Liu Z. (2019). Construction and application of regional business environment evaluation index system—A case study of Yangtze River economic belt. Financ. Econ..

[28] Feng R., Chu G., He M. (2019). Research on the construction of business environment index system in provinces along China’s “one belt and one road”. J. Changjiang Univ. Soc. Sci. Ed..

[29] Wei M., Li S. (2018). Measurement of China’s economic high quality development level in the new era. J. Quant. Tech. Econ..

[30] Zhang S., Kang B., Zhang Z. (2020). Evaluation of business environment in China: Index system and quantitative analysis. Econ. Manag..

[31] Sun Q., Chen H. (2020). Determinants, effects and evaluation indexes of regional business environment in China—Based on MIMIC model. Fisc. Res..

[32] Li Y., Zhang Z. (2019). Performance evaluation and optimization of listed forestry companies in China—Based on grey relational index screening and data envelopment analysis. For. Econ..

[33] Zhang J. (2019). Grey correlation analysis of agricultural processing industry and agricultural industrial structure in Qinzhou city. J. Anhui Agr. Sci..

[34] Bao J., Li Q., Fang B. (2019). Evaluation of industrial structure upgrading in Anhui province—Based on grey relational analysis. J. Tianjin Sino-Ger. Univ. Appl. Tec..

[35] Yang S., Li Y., Xu Q. (2019). Research on agricultural economic development and supply-side structural reform in Jilin province: Based on grey relational analysis. J. Chin. Agric. Mech..

[36] Zhong K.Y., Wang Y.F., Pei J.M., Tang S.M., Han Z.L. (2021). Super efficiency SBM-DEA and neural network for performance evaluation. Inf. Process. Manag..

[37] Zhong K., Li C., Wang Q. (2021). Evaluation of Bank Innovation Efficiency with Data Envelopment Analysis: From the Perspective of Uncovering the Black Box between Input and Output. Mathematics.

